# Organised chaos: entropy in hybrid inorganic–organic systems and other materials

**DOI:** 10.1039/c6sc02199a

**Published:** 2016-07-05

**Authors:** Keith T. Butler, Aron Walsh, Anthony K. Cheetham, Gregor Kieslich

**Affiliations:** a Centre for Sustainable Chemical Technologies , Department of Chemistry , University of Bath , Bath BA2 7AY , UK . Email: k.t.butler@bath.ac.uk; b WPI-Advanced Institute for Materials Research , Tohoku University , 2-2-1 Katahira , Aoba-ku, Sendai 980-8577 , Japan; c Department of Materials Science and Metallurgy , University of Cambridge , 27 Charles Babbage Road , Cambridge CB3 0FS , UK . Email: gk354@cam.ac.uk

## Abstract

Entropy is one of the fundamental quantities which links emerging research areas like flexibility and defect engineering in inorganic–organic hybrid materials. Additionally, a delicate balance between entropy and enthalpy can lead to intriguing temperature-driven transitions in such materials. Here, we briefly overview traditional material design principles, highlight the role of entropy in the past and discuss how computational methods can help us to understand and quantify entropic effects in inorganic–organic hybrid materials in the future.

## Abstract 2

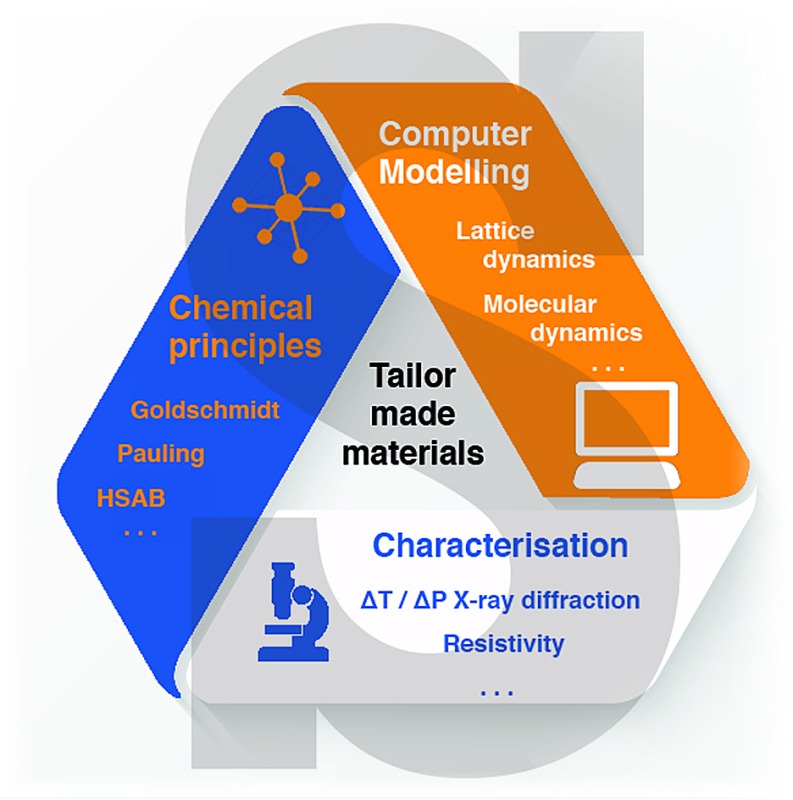
Entropy is one of the fundamental quantities which links emerging research areas like flexibility and defect engineering in inorganic–organic hybrid materials. Here, we highlight the role of entropy in the past and discuss how computational methods can help us to understand entropic effects in inorganic–organic hybrid materials in the future.

Since the beginning of modern crystal chemistry, the ultimate goal of materials science has been the design of materials with distinct physical properties. In this pursuit, many intriguing materials and systems with complex stimuli-signal behaviour have been prepared.^1–3^ For instance, Kitagawa *et al.* reported on the gas separation of CO_2_ and H_2_C_2_ by using (inverse) sorption selectivity in metal–organic frameworks,^4^ and Lotsch *et al.* described the preparation of layered systems in which the photonic properties vary upon changes in humidity.^5^ The complexity of the underlying structure–property-relationships, however, makes the targeted design of materials with specific properties challenging. This becomes even more complex for composite systems where the properties of two different materials are interconnected with each other.

Taking one step back and looking at more fundamental guidelines, some early milestone were the works by Goldschmidt,^6^ Pauling^7^ and Baur.^8^ In their studies, they approached the question ‘*under which circumstances a certain crystal structure forms*’ and established the first important guidelines in crystal chemistry. Factors such as packing density, coordination numbers and connectivity of polyhedra were identified. Together with the Hard and Soft Acid and Bases (HSAB) theory of Pearson,^9^ as well as sources of tabulated data, such as the Shannon ionic radii,^10^ solid state chemists possess a powerful tool-kit. These concepts can then be used to predict the existence and properties of many materials, and moreover, how properties can be altered in existing compounds. A simple and topical example is the decreasing band-gap of [CH_3_NH_3_]PbX_3_ along the halide series X = Cl^–^, Br^–^ and I^–^.^11^ The decreasing electronegativity of the halide anion leads to an increased band dispersion and therefore to a smaller band-gap. More examples of various materials can be found in the elaborate works by Hoffman,^12^ Goodenough^13^ and Whangbo^14^ amongst others.

Over the course of time, concepts emerged within different sub-areas of materials science that guided the development of new systems. Powerful and intuitive examples are the Goodenough–Kanamori rules for magnetism,^15,16^ the Schockley–Queisser limit for solar cells^17^ and the ‘phonon-glass electron-crystal’ model by Slack for thermoelectrics.^18^ In the field of inorganic–organic frameworks, ‘*the art of reticular synthesis*’, a rigid-body approach, has guided many experimental scientists in the preparation of new frameworks.^19^ In parallel, the foundation of modern density functional techniques was laid by Kohn and co-workers.^20,21^ In the latter part of the 20^th^ century, predictions of crystal structures based on judiciously parameterised empirical potentials became common.^22^ With the exponential growth in computer power, as predicted by Moore's law, fully *ab initio* methods based on density functional theory (DFT), as well as quantum chemical wavefunction methods, are now able to screen potential energy surfaces for local and global minima.^23^ All these approaches have in common that the factor of temperature, in particular entropy, is only qualitatively discussed. This does not mean that the role of entropy is generally underestimated, quite the contrary, since the impact of entropy is well known and in fact has sparked several research areas in the past.^24^

## Entropic effects in crystalline materials

The entropy of a perfect crystal is zero at 0 K. For inorganic materials, engineering entropy is closely related to defect chemistry and site disorder.^24^ The minimum in Gibbs free energy of a system is determined by a delicate balance between enthalpy and entropy at finite temperatures, this drives the formation of intrinsic defects. Such defects, more precisely their concentration, can be used to alter properties such as the ionic conductivity in solid state electrolytes, *e.g.* CaO or Y_2_O_3_ stabilised ZrO_2_.^25^ A further intriguing example is the defect chemistry of Fe_1–*x*_O at temperatures above 540 °C where specific defect cluster have been found, *e.g.* four Fe^2+^ defect sites surrounding a tetrahedral Fe^3+^.^26^ Here entropy leads to the formation of defects at high temperatures, whereas cluster formation is driven to enhance coulombic interactions. Site disorder, for example in alloys, is a source for configurational entropy and oftentimes challenging to assess. A stunning example in which disorder is paired with defect chemistry is Cu_2–*x*_Se.^27^ In this compound, Se atoms form a rigid face-centred cubic lattice, whereas copper ions are highly disordered with liquid-like mobilities.

Configurational entropy can determine the balance between ordered and disordered mixed valence systems across temperature ranges.^28^ Vibrational entropy on the other hand is closely related to the chemical bond organisation. In general, rigid inorganic lattices where high bond strengths are present suppress a high contribution from (vibrational) entropy. The role of vibrational entropy is therefore enhanced in Zintl compounds or inorganic clathrates where loosely bound (molecular) moieties exist. In Zn_4_Sb_3_ for example, isolated Sb_2_^4–^ dumbbells are responsible for phonon scattering, and also further contribute to vibrational entropy.^29^ Such ‘rattling-features’, similarly present in filled skutterudites A_B_Co_4_Sb_12_,^30^ have recently been explored in the context of thermoelectric research. Other examples where entropy plays a major role include temperature dependent phenomena, for instance, the driving factor for increasing crystal symmetry with temperature that is observed for most materials.^31^

Similarly, in the field of organic molecular crystals there is also intense interest in the prediction and understanding of structural polymorphism for applications as diverse as pharmaceuticals, electronic polymers and explosives.^32^ In molecular crystals the energy landscape frequently features several possible crystal structures with small energy differences between them.^33^ In many studies of packing in molecular crystals the free energy is approximated by the static lattice energy, neglecting the contribution from entropy. In some cases this may be justified; however, recent computational studies have highlighted the importance of vibrational entropy^32,34–36^ as well as configurational disorder.^37–39^ For instance, in molecular crystals vibrational entropy is found to be important in phase transitions of bistable materials^34^ and for preferential stabilisation of the predominant phase of aspirin.^35^

Moving onwards to hybrid inorganic–organic materials, the focus of this perspective, the situation becomes more intricate. For example, the name ‘metal–organic frameworks’ highlights the presence of organic and inorganic moieties in one framework. This gives the experimentalist a lot of freedom, though at the same time causes further challenges, *e.g.* computationally and from the rational design point of view. The more complex chemical patterns, in particular the variety of different bond strengths, intensifies the role of entropy. For example, rotational freedom is accessible for organic groups, *e.g.* the CH_3_CH_2_-backbone in BaCa_2_(CH_3_CH_2_COO), and can lead to dynamic behaviour and temperature-dependent phase transitions.^40^ A further important aspect is the possible formation of hydrogen bonds that are absent for inorganic materials. Such interactions, weak in comparison to ionic or covalent bonds between atoms, have a large impact in hybrid materials and often crystal structures are formed where hydrogen bonding interactions are maximised. In [(CH_3_)_2_NH_2_]Zn(HCOO)_3_, for example, a dense metal–organic framework (MOF), hydrogen bonds are responsible for a fascinating range of temperature dependent order–disorder phase transitions;^41^ in other words, the threshold temperature *T*_CRIT_ is accessible where *T*_CRIT_Δ*S*_DIS_ ≥ Δ*H*_HB_. In this example Δ*S*_DIS_ is the entropy gain through the disorder of [(CH_3_)_2_NH_2_]^+^ and Δ*H*_HB_ the enthalpy gain of ordered hydrogen bonds. The low density of porous systems, present in MOFs such as zeolitic imidazolate frameworks,^42^ further emphasises the role of entropy. These low densities usually lead to relatively high vibrational entropies (thermal displacements) and consequently to higher flexibility. It is therefore no surprise that recently a porous–nonporous phase transition has been observed in ZIF-4 upon cooling.^43^ The (thermo-responsive) breathing-behaviour of pillared-layered MOFs can also be interpreted within this context.^44,45^ Lastly, defects in hybrid materials, and in particular metal–organic frameworks, have recently gained much attention and further contribute to configurational entropy.^46^ Extensive reviews that focus on the classification, engineering and impact of defects in hybrid materials have recently appeared.^47,48^ Currently it seems that the impact of defects, or the phenomena associated with them, such as active catalytic sites in porous frameworks, are possibly even greater than in purely inorganic materials. However, it should be mentioned here, that nonstoichiometry as present in Fe_1–*x*_O, is virtually absent in hybrid materials. It normally requires mixed valance and is stabilised at high temperatures, which are inaccessible for hybrid materials. In general, mixed valence is accessible in stoichiometric hybrid frameworks such as [(CH_3_)_2_NH_2_][Fe^II^Fe^III^](HCOO)_6_ and [Li_*x*_Fe_*x*_^II^Fe_1–*x*_^III^(OH)_0.8_F_0.2_(O_2_CC_6_H_4_CO_2_)].^49,50^

All these effects together lead to a shallow energy surface in hybrid materials and accentuate the effect of usually weaker interactions, such as ligand field stabilisation energy, substituents of organic linkers and so on. The possibility of amorphisation and recrystallization of metal–organic frameworks is in strong agreement with this observation.^51,52^ It also seems that entropy is a basic principle for emerging research areas of flexibility and defects in hybrid materials. Many application-relevant properties are closely related to entropic effects, such as sorption properties in porous frameworks and temperature driven phase transitions, as well as the responses to external pressures for sensing applications, defect-driven carrier concentrations and mobilities, and thermal conductivities. Similar conclusions can be drawn for the field of porous organic cages.^53,54^ Despite the difficulties attendant with the estimation and computation of entropic effects, the task of understanding them is important enough to warrant serious research and further development in this area. Moreover, the growing availability of computational power means that the techniques for studying the effects of entropy can now be applied routinely to a wide range of hybrid systems.

## Computational aspects

As alluded to above, entropy in the solid-state can be broadly separated into three classes: (i) vibrational entropy, (ii) rotational entropy and (iii) configurational entropy. For metallic systems electronic entropy can also play an important role, but we restrict our discussion to materials with an electronic band gap. The theoretical basis for describing these effects in solid-state systems has been known for well over half a century. Starting from the harmonic oscillator model of a solid developed by Debye^55^ and Einstein,^56^ the lattice dynamics approach, formulated by Born,^57^ provides the necessary apparatus for the quantitative calculation of vibrations (phonon modes) and the vibrational contributions to crystal stability. Rotational effects on the other hand are almost absent in solely inorganic materials, where structural units, usually ions or atoms, are rotationally invariant. In hybrid materials, where molecules constitute an element of the structure, rotations become possible and can be accounted for through statistical mechanical approaches. Finally, in multi-component materials – for example double perovskites,^58,59^ mixed tetrahedral semiconductors or lattices with defect sites – disorder of site occupancy introduces the possibility of configurational entropy.

*Vibrational entropy* is computationally accessible *via* lattice dynamics calculations. Although the theory was formulated over half a century ago,^57^ it is only in recent years that the advent of high-performance computing has made the application of expensive first-principles calculations tractable. The basic premise starts from treating the system as a coupled harmonic oscillator. At the heart of the lattice dynamics method is the calculation of the dynamic matrix (**D**). **D** is obtained from the Hessian matrix. The Hessian contains the second derivatives of the energy with respect to the geometry of the system, which can be obtained directly from DFT calculations. Solving **D** yields a set of eigenvectors (phonon modes) and eigenvalues (phonon frequencies (*ω*)). The vibrational entropy of the system can be obtained from
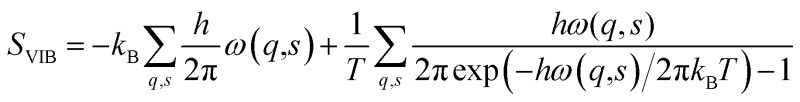
with *k*_B_ the Boltzmann constant, *h* the Planck constant, *T* the absolute temperature and *ω*(*q*,*s*) the phonon frequencies. The expense of the calculations arises from the fact that populating the Hessian requires many calculations to determine the forces on each ion. Additionally, it is necessary to use supercell expansions of the unit cell in order to ensure that all non-negligible elements of the Hessian are accounted for. The Hessian can be obtained in real space (finite displacement) or reciprocal space (perturbation theory). Most electronic structure packages provide the functionality to calculate the dynamical matrix. Additionally, several excellent post-processing packages have recently been developed,^60–62^ to obtain phonon spectra and interesting thermal properties, such as lattice expansion and thermal conductivity.

Looking at recent examples, DFT-based lattice dynamics has been applied to the calculation of vibrational entropies of hybrid formate systems. In such systems entropy plays a key role in determining which crystal polymorph is energetically favourable.^63^ Phonon modes are also of critical importance in the phenomenon of negative thermal expansion (NTE) in hybrid systems. Here lattice dynamics have been used to elucidate the microscopic origin of NTE.^64,65^ For instance, the exceptional NTE in MOF-5 is related to phonon softening due low frequency lattice modes of the benzenedicarboxylate linker and ZnO_4_ clusters. Furthermore, it is worth mentioning here, that lattice dynamics was also recently applied to explore the role of vibrational entropy in alloy formation.^66,67^

Interestingly, it had been assumed that vibrational entropy was not a significant factor in determining phase stability in molecular crystals.^68^ However, a recent computational assessment of 1061 such systems revealed that vibrational entropy contributions were large enough to re-rank polymorph stability in 9% of the pairs considered.^32^ This screening study used the lattice dynamics method, in association with a combined DFT and model potential energy functional. The model potential in this case was used to overcome the well-known limitations of DFT for considering dispersion interactions between molecules. Another similar approach employed DFT with a many-body dispersion correction, in association with lattice dynamics to predict the phase stability of aspirin.^35^

Lattice dynamics calculations also offer a powerful tool to simulate theoretical Raman and infra-red (IR) vibrational spectra of materials.^69–73^ Recently, the spectrum of the hybrid framework [CH_3_NH_3_]PbI_3_ has been calculated and compared to experimental data to allow for improved characterisation.^74^ Although the crystal vibrational contributions are expensive to calculate, more and more examples emerge that highlight the important nature of such effects, particularly in hybrid materials that exhibit soft phonon modes. These phonon modes emerge from weak interactions such as hydrogen bonds and are thermally accessible.

Molecular dynamics, MD, provides another route to exploring vibrational effects and has been used to demonstrate the impact of vibrational entropy in determining phase changes in organic polymer crystals.^34^ A recent study obtained the vibrational entropy of a radical magnetic molecular crystal as the difference between the internal energy of the system – obtained from MD simulations in canonical ensemble – and the Helmholtz free energy of the system – obtained by thermodynamic integration. This methodology is costly, as MD requires many thousands of steps to obtain well equilibrated quantities, whilst thermodynamic integration requires many (typically tens to hundreds) individual MD runs; however, in return one obtains quantities which transcend the (quasi-)harmonic approximation made in lattice dynamics techniques.

*Configurational entropy*, that is disorder in the nature of site occupancy on crystal sub-lattices, including defects, has long been known in solid-state chemistry. Standard simulation techniques employ periodic boundary conditions, where an infinite representation of the system is constructed from a repeating unit. This makes the explicit treatment of configurational disorder challenging, as there will always be a degree of periodicity in the system. In principle, choosing a large enough starting unit could account for disorder, but practically the size of unit is constrained by computational demands. To negate these issues, statistical thermodynamics methodologies for considering the entropies of ensembles of configurations have been developed. All of these methods start from the ideal entropy of mixing

*S*_MIX_ = –*nR*(*x*_1_ ln *x*_1_ + *x*_2_ ln *x*_2_)

where *n* is the number of particles and *x*_*n*_ are the fractions of each component. The ideal entropy of mixing assumes no inter-particle interactions. The effects on interatomic forces are then added using a variety of techniques. The most widely applied of these methods are the coherent potential approximation^75^ methods, the special quasi-random structure approach^76^ (SQS) and the cluster expansion method^77^ (CE). These have been used extensively to study the effects of disorder in solid-state systems. The coherent potential method suffers from its mean-field formulation when local structure effects become important, while the latter two explicitly treat structural variations and are available as part of the ATAT modelling package for atomistic simulations of disordered systems.^78^ Recently, a method called the ‘small set of ordered structures’ (SSOS) has been reported to provide results of similar accuracy to the SQS and CE methods. This is particularly interesting as SSOS is based on a smaller and more tractable set of calculations, allowing systems of up to quinary composition to be treated.^79^

The effects of configurational entropy have been studied for some time. In 1968 Navrotsky and Kleppa demonstrated the role of configurational entropy in determining normal-to-inverse cation ordering in spinels. With considering the thermodynamics of site occupancy disorder in ZnAlFeO_4_, Mg_2_TiO_4_, Co_2_TiO_4_, Zn_2_TiO_4_, CoZnTiO_4_, ZnNiTiO_4_, Co_2_GeO_4_, and Ni_2_GeO_4_ the authors demonstrate that spinels with random or highly inverse cation distributions have high configurational entropies and are favoured at higher temperatures.^80^ In the past decade, multiternary systems dubbed high-entropy alloys (HEAs) have been the subject of intense research. In the area of oxides, it was recently demonstrated that disorder on the cation sub-lattice of an equimolar mixture of MgO, CoO, NiO, CuO and ZnO can be stabilised by configurational entropy, resulting in new materials.^81^

Configurational entropy has largely been overlooked in molecular crystals. However, 20% of all structures in the Cambridge Structural Database of organic crystals report disorder.^38^ In some cases disorder has been accounted for by performing Monte Carlo (MC) calculations, including special move classes to probe dynamic phases.^37^ More recently, the generalised quasi-chemical approach (GQCA) has been applied to the study of disorder in eniluracil.^38^ This method used the SOD code^82^ to generate all symmetrically inequivalent configurations and then applied statistical mechanics to obtain free energies and entropies. It has been shown that such symmetry adapted ensemble approaches can predict the relative importance of disorder in determining the thermodynamic ground state of a crystal.^39^

In hybrid systems an understanding of configurational order/disorder relationships is critical in a number of scenarios. By mixing cations in MOFs it is possible to tune magnetic,^83^ catalytic^84^ and dynamic^85^ properties. In lead-based hybrid halide perovskites with the general formula APbX_3_ (A = [CH_3_NH_3_]^+^ or [NH_2_CHNH_2_]^+^ and X = Br^–^ or I^–^), some of the best efficiency solar cells are now obtained by mixing of organic cations on the A-site and halides on the X site.^86^ Recently DFT calculations have shown the complexity of the Br/I solid solution [CH_3_NH_3_]PbI_1–*x*_Br_*x*_, which demonstrates the spinodal decomposition of a mixed halide solution into iodide and bromide rich phases at room temperature.^87^ Engineering defects in MOFs is another area of growing importance and there has been a flurry of interest in harnessing the effects of defects to tune materials properties.^47,48,88^ For example, in HKUST-1 with careful linker selection, properties such as porosity and band-gap can be gradually tuned and computationally rationalised.^89^ Although this area is only starting to emerge, the importance of configurational entropy cannot be overestimated. In all of these systems, the configurational entropy will be key in driving systems between correlated and un-correlated impurity/defect centres, as such calculations are expected to play a crucial role in the design of hybrid solid-solutions and defects.

*Rotational entropy* is seldom a concern in solid-state sciences given the spherical symmetry of constituent building blocks (ions). Consequently, theories tackling contributions from rotational entropy in the solid-state are less developed. When rotational entropy is considered it is generally in the form of the addition of an analytical term to the energy after a total energy calculation. The analytical expression for rotational entropy of a molecular unit is
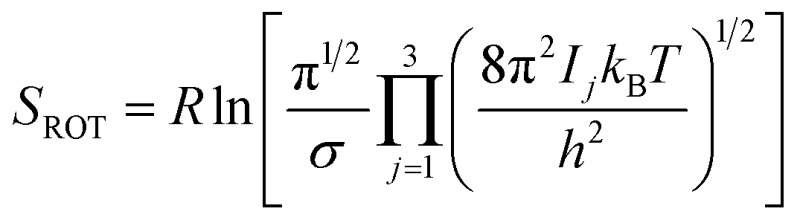
where *I*_*j*_ are the principle moments of inertia of the molecule and *σ* is the symmetry number. In soft-matter simulations, however, rotational entropic effects have been shown to play important roles in phenomena such as water confinement in carbon nanotubes^90^ and protein–ligand interactions.^91^ Methods have been proposed for partitioning total entropy from molecular dynamics simulations to obtain a rotational contribution.^92^ Rotational disorder is also a key consideration in the study of “plastic crystals”, where molecular orientations are determined stochastically at finite temperatures. In plastic crystals a number of useful concepts have been developed for understanding the effects of rotational disorder, for example molecular pseudospins, which describe the orientation of a molecule in space, and rotator functions, which are functions that change continually with varying orientation.^93^ It is our strong feeling that in the field of hybrid materials the study of rotational entropic effects is an area of growing importance. For instance, rotationally driven order–disorder transitions lead to marked changes in dielectric properties.^2^ From the computational side, however, this is little understood and represents one of the future directions of this field, and much can be learned from the simulation of soft-matter systems (Table 1).

**Table 1:** Types of entropy, related properties and how they can be accounted for computationally

Entropy	Properties	Calculation
Configurational	Bandgap; magnetic ordering; thermal conductivity; phase stability; electronic polarisation	Cluster expansion; special quasirandom structures; small set of ordered structures; lattice Monte Carlo
Vibrational	Bandgap; phase stability; thermal conductivity; vibrational spectra	Lattice dynamics; molecular dynamics
Rotational	Phase stability; thermal conductivity; electrical polarisation	Molecular dynamics; nudged elastic bands

In spite of the progress to date, there are outstanding challenges in the areas of computational materials science, which must be addressed in order to capture all phenomena and to facilitate a truly predictive computational design of hybrid materials.

## Key challenges in the field of theory development

*Vibrational entropy*: going beyond the quasi-harmonic approximation. The current state-of-the-art in accounting for vibrational entropy from lattice dynamic calculations is the quasi-harmonic approximation. Thermal expansion is treated as a series of volume-dependent harmonic calculations. This neglects anharmonicity in the energy surface which can become important at higher temperature, as recently demonstrated for defect formation in Al and Cu.^94^ Higher-order anharmonic effects are essential for describing systems that exhibit displacive or order–disorder instabilities.

*Configurational entropy*: disorder of multi-component systems. The challenge here is largely implementational. Most tools developed for considering the effects of site disorder in materials deal with single atomic or ionic units. For hybrid systems it becomes necessary to consider disorder of molecular units, too; in this case a unit at a particular crystal site can change its effect by changing its orientation. These extra orientational degrees of freedom increase the combinatorial space to be considered, but will be important for obtaining quantitative values for hybrid systems. We note that methods developed for inorganic systems have been applied to molecular crystals^38,39^ and extensions to hybrid materials should not be arduous.

*Rotational entropy*: explicit calculations. As we stated above, the calculation of rotational entropy in solid-state hybrid systems is considered analytically, if at all. Direct calculation of rotational entropy requires the application of molecular dynamics (MD) calculations on a timescale long enough to capture the statistics of rotations. This can require many hundreds of thousands or even millions of steps, depending on the system. Typical MD time-steps are of the order of 1 fs, whilst rotations can occur on the timescale of pico- to nano-seconds.^41^ One possible route to obtaining accurate information on rotational entropy would be to apply biased-MD calculations.^95^ Such methods selectively drive the system along a specified variable, whilst maintaining the integrity of the underlying thermodynamic ensemble and actively promoting the occurrence of rare-events. Metadynamics and related methods are commonly used in fields such as protein folding,^96^ mineralisation^97,98^ and the exploration of nanoclusters.^99^ It is our belief that it could be a very useful tool for exploring phenomena such as rotational effects in hybrid materials. Moreover, the methods of pseudospins and rotator functions, mentioned previously, have recently been applied to understanding the role of rotational disorder in driving ferroelectric transitions in hybrid perovskite materials.^100^

A final important development in the area of computation, which we have not mentioned previously, is the application of high-throughput automated calculations and statistical (or machine) learning. Although phonon calculations are significantly more expensive than standard total energy calculations, high-throughput studies are beginning to be reported.^101^ The first libraries of computed phonon data are now available^102^ and facilitate the application of machine learning techniques. In any machine learning study the availability of good descriptors, simple properties which can be related to more complex outcomes of the systems, is critical.^103^ One factor suggested by Greer is the discrepancy in ion sizes in a multi-ternary system.^104^ A recent data-mining study drawing on empirical data^105^ suggested two descriptors – Ω and δ – the former based on entropy of mixing, the latter based on radii to predict high-entropy alloy formation. Another study, derived from calculated data, proposes a descriptor based on “thermodynamic density of competing crystalline states” to predict the formation of bulk metallic glasses.^106^ We have no doubt that data-mining techniques will become ever more important in the design of materials and the elucidation of the role of entropy.

## Entropy as design principle?

Undoubtedly, entropy has a tremendous impact on the crystal chemistry of hybrid inorganic–organic materials and includes all aspects of dynamic effects and structure–property relationships. Recent breakthroughs in the field have combined *chemical design principles*, *experimental observations* and *computational chemistry* with each other.^107,108^ From that viewpoint it is clear that only a common effort involving different research areas, and therefore research groups, is capable of fully unravelling the power of entropy as a design principle in the future.

In the majority of cases where entropy is considered in solid-state systems, only one of the three types we have outlined is considered. Examples exist, however, such as the defect dependent NTE in UIO-66,^109^ where a combination of configurational disorder together with vibrational entropy account for the material's properties. The first step towards an elaborate understanding of entropy is the analysis of each contribution and its importance within the system as a whole. A recent example where combined efforts lead to a deep understanding is the solar cell material [CH_3_NH_3_]PbI_3_. At a first glance, the system seems to be rather simple, but [CH_3_NH_3_]PbI_3_ has proven to exhibit a highly dynamic inorganic framework.^110,111^ In these hybrid perovskite systems, more specifically their alloys, the important roles of both configurational^87^ and vibrational^74^ entropy have been emphasised by computational studies. Such studies provide important guidelines and methodological blueprints for future investigations into structure–property relationships in hybrid metal–organic systems.

In hybrid materials in general, it is well established that long organic linkers and porous materials lead to high vibrational entropies, while, at the same time, substitutional defects seem to be relatively easy to introduce in hybrid materials. For the impact of rotational entropy, the field of hybrid materials can learn from related research areas. For instance, the structure of helical polymers is mainly influenced by salt-bridge like interactions which have been designed accordingly.^112^ It is worth remembering here that the nearly unlimited variety of organic linker materials allow for the specific design of hydrogen bonds, van der Waals forces, π–π interactions and so on.

Ideally, computational chemistry approaches for hybrid systems will become as sophisticated as presently available methods for inorganic and soft-matter materials. As mentioned earlier, there are many aspects that need to be tackled in the future; however, experimental scientists are now creating a need for such theories. This can lead to fast and fruitful progress, as has been recently shown in the area of gas-sorption in metal–organic frameworks. The enormous interest in the materials science community, including within industry, has led to a need for computational models to describe such phenomena. As a consequence, computational codes were modified and guided the synthesis of new highly-porous gas-storage materials, aided by the use of high-throughput screening techniques.^113–115^ It is our belief that a similar demand for insight will lead to developments in the understanding of entropy in hybrid inorganic–organic materials.

## Conclusion

In conclusion, we have discussed the role of structural and chemical disorder in the context of hybrid materials chemistry. We have outlined three primary contributions to entropy in metal–organic systems and have given examples of how these are related to current developments in the field of hybrid inorganic–organic materials. In particular, focus has been laid on the developments and key challenges in the field of computational chemistry.

50 years after R. H. Fowler stated that “*[t]here is no hope for a logical definition of absolute entropy*”,^116^ referring to the endless possible sources of entropy, significant progress has been made in enumerating and understanding the important sources of entropy and helped to understand experimental findings. Thus we believe that the progress made can be used to understand and engineer the physical and chemical properties in hybrid inorganic–organic materials in the future. It will be exciting to see if in the future a combined approach of *computational chemistry*, *experimental approaches* and *chemical design principles* can help to ‘find’ the philosopher's stone of materials science – the targeted design of materials with distinct physical properties.
